# Association of Occupational Exposure to Disinfectants With Incidence of Chronic Obstructive Pulmonary Disease Among US Female Nurses

**DOI:** 10.1001/jamanetworkopen.2019.13563

**Published:** 2019-10-18

**Authors:** Orianne Dumas, Raphaëlle Varraso, Krislyn M. Boggs, Catherine Quinot, Jan-Paul Zock, Paul K. Henneberger, Frank E. Speizer, Nicole Le Moual, Carlos A. Camargo

**Affiliations:** 1 INSERM U1168, VIMA: Aging and Chronic Diseases, Epidemiological and Public Health Approaches, F-94807, Villejuif, France; 2 University de Versailles St-Quentin-en-Yvelines, UMR-S 1168, F-78180, Montigny le Bretonneux, France; 3 Channing Division of Network Medicine, Department of Medicine, Brigham and Women’s Hospital and Harvard Medical School, Boston, Massachusetts; 4 Department of Emergency Medicine, Massachusetts General Hospital and Harvard Medical School, Boston, Massachusetts; 5 Barcelona Institute for Global Health (ISGlobal), Barcelona, Spain; 6 Universitat Pompeu Fabra (UPF), Barcelona, Spain; 7 CIBER Epidemiología y Salud Pública (CIBERESP), Madrid, Spain; 8 Respiratory Health Division, National Institute for Occupational Safety and Health, Morgantown, West Virginia

## Abstract

**Importance:**

Exposure to disinfectants in health care workers has been associated with respiratory health outcomes, including asthma. Despite the biological plausibility of an association between disinfectants (irritant chemicals) and risk of chronic obstructive pulmonary disease (COPD), available data are sparse.

**Objective:**

To investigate the association between exposure to disinfectants and COPD incidence in a large cohort of US female nurses.

**Design, Setting, and Participants:**

The Nurses’ Health Study II is a US prospective cohort study of 116 429 female registered nurses from 14 US states who were enrolled in 1989 and followed up through questionnaires every 2 years since. The present study included women who were still in a nursing job and had no history of COPD in 2009, and used data from the 2009 through 2015 questionnaires. Clean and complete data used for this analysis were available in July 2018, and analyses were conducted from September 2018 through August 2019.

**Exposures:**

Occupational exposure to disinfectants, evaluated by questionnaire and a job-task-exposure matrix (JTEM).

**Main Outcomes and Measures:**

Incident physician-diagnosed COPD evaluated by questionnaire.

**Results:**

Among the 73 262 women included in the analyses, mean (SD) age at baseline was 54.7 (4.6) years and 70 311 (96.0%) were white, 1235 (1.7%) black, and 1716 (2.3%) other; and 1345 (1.8%) Hispanic, and 71 917 (98.2%) non-Hispanic. Based on 368 145 person-years of follow-up, 582 nurses reported incident physician-diagnosed COPD. Weekly use of disinfectants to clean surfaces only (16 786 [22.9%] of participants exposed) and to clean medical instruments (13 899 [19.0%] exposed) was associated with COPD incidence, with adjusted hazard ratios of 1.38 (95% CI, 1.13-1.68) for cleaning surfaces only and 1.31 (95% CI, 1.07-1.61) for cleaning medical instruments after adjustment for age, smoking (pack-years), race, ethnicity, and body mass index. High-level exposure, evaluated by the JTEM, to several specific disinfectants (ie, glutaraldehyde, bleach, hydrogen peroxide, alcohol, and quaternary ammonium compounds) was significantly associated with COPD incidence, with adjusted hazard ratios ranging from 1.25 (95% CI, 1.04-1.51) to 1.36 (95% CI, 1.13-1.64). Associations were not modified by smoking or asthma status (*P* for interaction > .15).

**Conclusions and Relevance:**

These longitudinal results suggest that regular use of chemical disinfectants among nurses may be a risk factor for developing COPD. If future studies confirm these results, exposure-reduction strategies that are compatible with infection control in health care settings should be developed.

## Key Points

**Question:**

Is exposure to disinfectants and cleaning products associated with incidence of chronic obstructive pulmonary disease among health care workers?

**Findings:**

In a cohort study of 73 262 US female nurses participating in the Nurses’ Health Study II who were followed up from 2009 to 2015, occupational exposure to cleaning products and disinfectants was significantly associated with a 25% to 38% increased risk of developing chronic obstructive pulmonary disease independent of asthma and smoking.

**Meaning:**

This study’s findings suggest that regular use of chemical disinfectants among nurses may be a risk factor for developing chronic obstructive pulmonary disease.

## Teaser

This cohort study evaluates data from the Nurses’ Health Study II to investigate the association of occupational exposure to disinfectants and other cleaning products with the incidence of chronic obstructive pulmonary disease (COPD) among female US registered nurses with no history of COPD at baseline.

## Introduction

Chronic obstructive pulmonary disease (COPD) is the third leading cause of mortality worldwide and among the diseases contributing the most to disability-adjusted life-years.^1^ Although tobacco smoke remains the major risk factor for COPD development in the United States and other industrialized countries, occupational exposures contribute substantially to the burden of disease.^2^ A growing body of data suggests that 15% to 20% of cases of COPD are attributable to occupational exposures.^3,4^ However, despite the general recognition of an association between occupational exposures and COPD,^2,5^ few individual causal agents have been identified.^5,6^ Most studies on occupation and COPD have investigated broad exposure categories (eg, “vapor, gases, dust, or fumes”), which include many agents.^7^ Industry-specific studies, which generally provide more insight regarding specific causal agents than population-based studies, have focused on a limited number of occupational settings, which are often occupations with predominantly male workers.^3^ Although chemical exposures are an important component of vapor, gases, dust, or fumes ^7^ and may account for a large part of COPD risk,^4,8^ their association with COPD remains unclear.^3^

Exposure to cleaning products and disinfectants is common at work and at home and remains more frequent among women.^9,10^ Exposure levels are particularly high in the health care industry,^11^ one of the largest employment sectors in the United States and Europe.^12^ The respiratory health risks associated with exposure to cleaning products and disinfectants are increasingly recognized.^13^ Although investigators have primarily focused on asthma,^13,14,15^ the irritant properties of many chemicals contained in disinfectants^13^ support the study of a broader range of respiratory effects. A few European studies have reported an increased risk of COPD,^16,17^ accelerated lung function decline,^9^ and higher rates of death due to COPD^18^ among cleaning workers. However, to our knowledge, no study to date has investigated the association of occupational exposure to disinfectants and cleaning products with COPD risk among health care workers, nor has any study suggested specific chemicals that may underlie the association between cleaning jobs and COPD.^19^ Determining specific agents associated with adverse health outcomes is crucial in health care settings. Indeed, prevention strategies are often based on avoidance of the causal agent(s); however, adequate levels of disinfection must be maintained in health care settings to protect patients and workers from nosocomial infections.^20^

Using data from the Nurses’ Health Study II (NHSII), a large, ongoing, prospective study of US female nurses, we investigated the association between exposure to disinfectants and cleaning products and risk of incident COPD.

## Methods

### Population

The NHSII began in 1989 when 116 429 female registered nurses from 14 US states, aged 25 to 44 years, completed a questionnaire about their medical history and lifestyle characteristics.^21^ Follow-up questionnaires have been sent every 2 years since. The NHSII was initially designed to study women’s health (eg, long-term health effects or oral contraceptive use; determinants of major chronic diseases in women, such as breast cancer or cardiovascular disease), and the range of risk factors and health outcome data collected has expanded over time.^22^ Information on occupational exposures was collected for the first time in 2009, which was defined as the baseline for the present study. This investigation was approved by the institutional review board at Brigham and Women’s Hospital, and participants provided written informed consent. This study followed the Strengthening the Reporting of Observational Studies in Epidemiology (STROBE) reporting guideline.

Among the 116 429 participants in the NHSII, 98 817 returned at least 1 biennial questionnaire during the follow-up period (2011-2015); of these, 76 331 were still in a nursing job (eFigure in the Supplement). Among these respondents, we selected women without missing data for occupational exposure and pack-years of smoking who had not reported any history of COPD at baseline (2009). Clean and complete data used for this analysis were available in July 2018, and analyses were conducted from September 2018 through August 2019.

### Occupational Exposure to Disinfectants

Information on nursing job types (education or administration, outpatient/other nurses, emergency department or inpatient unit, and operating room) and general disinfection tasks (frequency of use of disinfectants to clean surfaces/medical instruments) was collected by questionnaire in 2009, 2011, and 2013.^23^ Information on the use of sprays (for instrument cleaning/disinfection, surface cleaning/disinfection, patient care, air refreshing, or other) was collected in 2011 and 2013. Disinfection tasks were studied using a dichotomous (weekly use of disinfectants to clean surfaces or medical instruments vs less than weekly) and a 3-level (no disinfection task performed weekly, weekly use of disinfectants to clean surfaces only, or weekly use of disinfectants to clean medical instruments regardless of the use of disinfectants to clean surfaces) variable. Frequency of cleaning or disinfection tasks and spray use (never, <1 d/wk, 1-3 d/wk, or 4-7 d/wk) was also examined.

Exposure to 7 of the most commonly used disinfectants or cleaning products (formaldehyde, glutaraldehyde, hypochlorite bleach, hydrogen peroxide, alcohol, quaternary ammonium compounds, and enzymatic cleaners) was evaluated by a nurse-specific job-task-exposure matrix (JTEM), as described in detail elsewhere^24^ and in the eAppendix in the Supplement. The JTEM assigned exposure level (low, medium, or high) based on types of nursing jobs and general disinfection tasks. Exposure to specific disinfectants according to the JTEM was thus studied using 3-level variables, with separate models for each disinfectant. Then, because nurses were often classified as exposed to multiple products, we studied exposure to combinations of several specific products evaluated by the JTEM^14^ for the products found associated with COPD when studied separately.

### COPD Incidence

In biennial questionnaires, participants were asked to report any condition(s) with which they were diagnosed since the last questionnaire cycle, including emphysema or chronic bronchitis. We used this information to identify incident cases of physician-diagnosed COPD (primary case definition) from 2009 to 2015.

Between October 2015 and December 2017, we sent a supplemental questionnaire on COPD to participants who reported a physician’s diagnosis of emphysema or chronic bronchitis in any past biennial questionnaire. Based on information collected on the supplemental questionnaire, we selected participants who reiterated that a physician had diagnosed them as having emphysema, chronic bronchitis, COPD, or any combination of these. This definition (stringent case definition), validated^25^ in a random sample of participants with COPD in NHSII (eAppendix in the Supplement), was used in a sensitivity analysis.

### Statistical Analysis

Prospective associations between occupational exposures and COPD incidence were evaluated by using Cox proportional hazards regression models. In each model, occupational exposure was handled as a time-varying variable and was evaluated as the highest exposure level at any of the questionnaire cycles before time of diagnosis. All Cox proportional hazards regression models were stratified by age and calendar year. For race and ethnicity, multiple categories were combined to create binary general categories. Analyses were adjusted for race (white vs black and other), ethnicity (Hispanic vs non-Hispanic), smoking habits (nonsmoker, ex-smoker, or current smoker), pack-years of smoking, and body mass index (calculated as weight in kilograms divided by height in meters squared into categories of <25.0, 25.0-29.9, and ≥30.0). Several sensitivity analyses were conducted, further adjusting the models for diet quality as measured by the Alternate Healthy Eating Index 2010,^26^ divided into quintiles, or for pack-years^2^ of smoking and excluding participants with previous comorbidities (cardiovascular diseases and cancer). We performed stratified analyses by smoking status, asthma status, and job change (any change in job type from the 2009 to 2013 questionnaires) and tested interactions in multivariable models. We estimated the population-attributable fraction of weekly use of any disinfectant on COPD risk among female nurses as PAF = pc(1 − 1/AHR), in which PAF is the population-attributable fraction; pc, the prevalence of exposure among cases of COPD; and AHR, the adjusted hazard ratio.^27^ A 2-sided *P* < .05 was considered statistically significant. Analyses were conducted using SAS, version 9.4 (SAS Institute Inc).

## Results

Among the 73 262 women who were eligible for analysis, mean (SD) age at baseline (in 2009) was 54.7 (4.6) years; 70 311 (96.0%) were white, 1235 (1.7%) black, and 1716 (2.3%) other; 1345 (1.8%) were Hispanic and 71 917 (98.2%) non-Hispanic; 4162 (5.7%) were current smokers and 20 631 (28.2%) were former smokers. Regarding cleaning/disinfection tasks, 16 786 (22.9%) of the nurses reported weekly use of disinfectants to clean surfaces only, and 13 899 (19.0%) reported weekly use of disinfectants to clean medical instruments. Very small but statistically significant differences were seen in sociodemographic characteristics according to disinfection tasks (Table 1); in particular, nurses reporting weekly use of disinfectants were younger and more often former or current smokers.

**Table 1.** Baseline Characteristics of the Study Population According to Disinfectant Use Among 73 262 US Female Nurses

Population Characteristics^a^	Weekly Use of Disinfectants to Clean Surfaces and/or Instruments			*P* Value
	None (n = 42 577)	Surface Only (n = 16 786)	Instruments (n = 13 899)	
Age, mean (SD), y	55.0 (4.6)	54.5 (4.6)	54.1 (4.6)	<.001
Race				
White	40 908 (96.1)	16 120 (96.0)	13 283 (95.6)	<.001
Black	746 (1.7)	274 (1.6)	215 (1.5)	
Other	923 (2.2)	392 (2.3)	401 (2.9)	
Ethnicity				
Hispanic	761 (1.8)	338 (2.0)	246 (1.8)	.15
Non-Hispanic	41 816 (98.2)	16 448 (98.0)	13 653 (98.2)	
Smoking status				
Never smoker	28 050 (65.9)	11 286 (67.2)	9129 (65.7)	<.001
Former smoker	12 290 (28.9)	4514 (26.9)	3827 (27.5)	
Current smoker	2233 (5.2)	986 (5.9)	943 (6.8)	
Pack-years of cigarette smoking among ever smokers, mean (SD)	13.6 (11.9)	13.4 (11.6)	14.2 (12.3)	.05
BMI at baseline^b^				
<20.0	1900 (4.6)	750 (4.7)	590 (4.4)	.02
20.0 to 24.9	14 583 (35.5)	5641 (35.2)	4596 (34.5)	
25.0 to 29.9	12 512 (30.5)	4878 (30.4)	4270 (32.1)	
≥30.0	12 101 (29.4)	4764 (29.7)	3853 (29.0)	
Job type				
Education or administration	11 085 (26.0)	1431 (8.5)	588 (4.2)	<.001
Outpatient, other nurses	23 714 (55.7)	8945 (53.3)	6768 (48.7)	
ED or inpatient unit	6647 (15.6)	4830 (29.4)	5104 (36.7)	
Operating room	1131 (2.7)	1580 (9.4)	1439 (10.4)	

Abbreviations: BMI, body mass index (calculated as weight in kilograms divided by height in meters squared); ED, emergency department.

^a^ Data are presented as number (percentage) unless otherwise stated. Values of categorical variables may not total 100% because of rounding.

^b^ There were 2824 missing values (3.8%) for BMI.

### Cleaning/Disinfection Tasks and COPD Incidence

Based on 368 145 person-years of follow-up from 2009 to 2015, 582 nurses reported incident physician-diagnosed COPD. In multivariable models, no single nursing job type was associated with COPD incidence, although a significant trend toward an increased risk of COPD incidence was observed when classifying jobs according to the level of disinfectant use (Table 2). Weekly use of any disinfectants was positively associated with COPD incidence, with an adjusted hazard ratio (AHR) of 1.35 (95% CI, 1.14-1.59). The corresponding population-attributable fraction (calculated in combination with a prevalence of exposure among cases of COPD of 45%) was 12%. Associations were observed for use of disinfectants both to clean surfaces only (AHR, 1.38; 95% CI, 1.13-1.68) and to clean medical instruments (AHR, 1.31; 95% CI, 1.07-1.61). When we examined the frequency of cleaning/disinfection tasks (Figure), risk of COPD incidence was highest among nurses with the greatest frequency of use (4-7 d/wk). A significant dose-response association was observed for the use of any disinfectants (for frequency of 4-7 d/wk: AHR, 1.43; 95% CI, 1.13-1.80; *P* for trend < .001) and disinfectants to clean surfaces (for frequency of 4-7 d/wk: AHR, 1.37; 95% CI, 1.09-1.72; *P* for trend, .003). Weekly use of spray (vs less than weekly) was not significantly associated with COPD incidence. However, when we examined frequency of spray use, a significant trend toward an increased risk of COPD incidence with higher frequency of use was observed (*P* for trend, .03), with a dose-response association.

**Table 2.** Prospective Association of Job Types and Self-reported Cleaning/Disinfection Tasks With Chronic Obstructive Pulmonary Disease Incidence Among US Female Nurses

Occupational Exposure^a^	Person-Years	No. of Cases	Age-Adjusted HR (95% CI)	*P* for Trend	Multivariable-Adjusted HR (95% CI)^b^	*P* for Trend
Job type^c^						
Education or administration	52 909	85	1 [Reference]	.004	1 [Reference]	.02
Outpatient, other nurses	196 143	285	0.99 (0.78-1.26)		1.03 (0.81-1.32)	
ED or inpatient unit	95 095	168	1.32 (1.01-1.71)		1.24 (0.95-1.62)	
Operating room	23 998	44	1.36 (0.94-1.96)		1.38 (0.96-1.99)	
Weekly use of disinfectant						
None	183 480	276	1 [Reference]	NA	1 [Reference]	NA
Any disinfectant	184 665	306	1.35 (1.14-1.59)		1.35 (1.14-1.59)	
Surface only	93 443	161	1.35 (1.11-1.65)		1.38 (1.13-1.68)	
Instruments	91 222	145	1.35 (1.10-1.65)		1.31 (1.07-1.61)	
Weekly use of sprays^d^						
No	196 903	214	1 [Reference]	NA	1 [Reference]	NA
Yes	56 702	75	1.31 (1.00-1.70)		1.27 (0.97-1.66)	

Abbreviations: ED, emergency department; HR, hazard ratio; NA, not applicable.

^a^ Exposure was evaluated as the highest exposure level at any of the questionnaire cycles before time of diagnosis. Follow-up periods were 2009 to 2015 for job type and use of disinfectants (368 145 person-years; 582 cases) and 2011 to 2015 for use of sprays (253 606 person-years; 289 cases). Job type, weekly use of disinfectant, and weekly use of spray were evaluated in separate models.

^b^ Multivariable models were adjusted for age, smoking status and pack-years (continuous), race, ethnicity, and body mass index. Observations with missing values for pack-years of smoking (<0.5%) were excluded from analyses. Observations with missing values for body mass index (3.8%) were included in the model as a “missing” category.

^c^ Job types are classified in increasing order of frequency of disinfectant use, as shown previously.^23^

^d^ Use of sprays for patient care, instrument cleaning or disinfection, surface cleaning or disinfection, air refreshing, or other.

**Figure.**
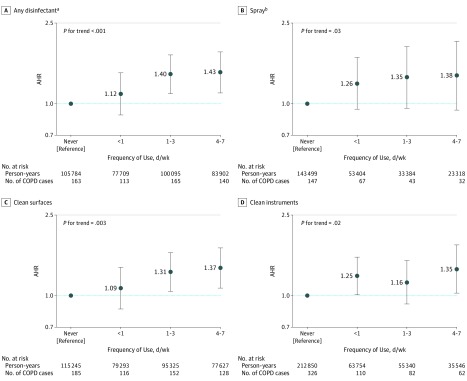
Prospective Association Between Self-reported Frequency of Cleaning/Disinfection Tasks and Chronic Obstructive Pulmonary Disease (COPD) Incidence Among US Female Nurses Occupational exposure was evaluated as the highest exposure level at any of the questionnaire cycles before time of diagnosis. The follow-up periods were 2009 to 2015 for job type and use of disinfectants and 2011 to 2015 for use of sprays. Multivariable models were adjusted for age, smoking status and pack-years (continuous), race, ethnicity, and body mass index. Observations with missing values for pack-years of smoking (<0.5%) were excluded from analyses. Observations with missing values for body mass index (3.8%) were included in the model as a “missing” category. Error bars indicate 95% CIs. Adjusted hazard ratios (AHRs) with 95% CIs are shown for type of disinfectant (A and B) and specific use of disinfectant (C and D). ^a^Use of a disinfectant to clean surfaces or instruments. ^b^Use of sprays for patient care, instrument cleaning or disinfection, surface cleaning or disinfection, air refreshing, or other.

Results were similar in sensitivity analyses excluding participants with previous comorbidities (cardiovascular diseases and cancer) or in models further adjusted for pack-years^2^ of smoking or diet quality (eTable 1 in the Supplement). Moreover, the association between weekly use of any disinfectants and COPD incidence was significant and similar in never smokers (AHR, 1.34; 95% CI, 1.03-1.73; eTable 2 in the Supplement) and ever smokers (AHR, 1.38; 95% CI, 1.11-1.71; *P* for interaction = .91) and in participants with asthma (AHR, 1.37; 95% CI, 1.02-1.84; eTable 3 in the Supplement) and without asthma (AHR, 1.31; 95% CI, 1.03-1.66; *P* for interaction = .78). When we examined the types of disinfection tasks, the association between use of disinfectants to clean surfaces only and COPD incidence was more pronounced among ever smokers and participants without asthma, whereas the association between use of disinfectants to clean medical instruments and COPD incidence was more pronounced among never smokers and participants with asthma. Nonetheless, the latter differences by smoking or asthma status were not statistically significant (all *P* for interaction > .15). The association between weekly use of any disinfectants and COPD incidence was also similar in participants who changed nursing job type between 2009 and 2013 (47 788 [65%]; AHR, 1.41; 95% CI, 1.14-1.73) and participants who did not (25 474 [35%]; AHR, 1.33; 95% CI, 1.00-1.78; *P* for interaction = .52). In the sensitivity analysis using a more stringent COPD definition (eTable 4 in the Supplement), the association between use of disinfectants to clean surfaces only and COPD incidence was similar (AHR, 1.32; 95% CI, 0.99-1.78; *P* = .06), although statistical power was no longer significant owing to fewer cases of COPD. In contrast, the association between use of disinfectants to clean instruments and COPD incidence was no longer significant (AHR, 1.14; 95% CI, 0.83-1.56; *P* = .41).

### Specific Disinfectants/Cleaning Products Evaluated by JTEM and COPD Incidence

In multivariable models using the JTEM estimates (Table 3), no statistically significant association was observed between high-level exposure to enzymatic cleaners (AHR, 1.05; 95% CI, 0.84-1.31) or formaldehyde (AHR, 1.20; 95% CI, 0.92-1.57) and COPD incidence. In contrast, high-level exposure to glutaraldehyde, bleach, hydrogen peroxide, alcohol, and quaternary ammonium compounds was significantly associated with increased risk of COPD incidence, with AHRs ranging from 1.25 (95% CI, 1.04-1.51) to 1.36 (95% CI, 1.13-1.64). No significant association was observed when we compared medium- and low-exposure level (AHRs ranged from 1.18 [95% CI, 0.95-1.46] to 1.32 [95% CI, 0.98-1.79]) except for glutaraldehyde (AHR, 1.50; 95% CI, 1.18-1.90).

**Table 3.** Prospective Associations Between Exposure to Specific Disinfectants/Cleaning Products Evaluated by Job-Task-Exposure Matrix and Chronic Obstructive Pulmonary Disease Incidence Among US Female Nurses

Occupational Exposure^a^	Person-Years	No. of Cases	Multivariable-Adjusted HR (95% CI)^b^
Formaldehyde	38 056	62	1.20 (0.92-1.57)
Glutaraldehyde	125 281	192	1.25 (1.04-1.51)
Hypochlorite bleach	127 879	215	1.36 (1.13-1.64)
Hydrogen peroxide	141 504	229	1.29 (1.08-1.54)
Alcohol	150 512	245	1.32 (1.10-1.59)
Quaternary ammonium compounds	142 722	233	1.33 (1.11-1.60)
Enzymatic cleaners	69 447	97	1.05 (0.84-1.31)

Abbreviation: HR, hazard ratio.

^a^ Exposure was evaluated as the highest exposure level at any of the questionnaire cycles before time of diagnosis. Associations presented compare high-exposure level vs low-exposure level for each product. Observations with missing values for pack-years of smoking (<0.5%) were excluded from analyses. Observations with missing values for body mass index (3.8%) were included in the model as a “missing” category.

^b^ Multivariable models were adjusted for age, smoking status and pack-years (continuous), race, ethnicity, and body mass index.

When we analyzed combinations of specific products (eTable 5 in the Supplement), no associations with COPD incidence were observed among nurses exposed to alcohol or quaternary ammonium compounds only or to aldehydes (formaldehyde or glutaraldehyde) but not to the other products. In contrast, a significantly increased risk of COPD incidence was observed among nurses exposed to hypochlorite bleach or hydrogen peroxide with or without aldehydes.

## Discussion

This analysis of a cohort of 73 262 US nurses followed up across 6 years showed that occupational exposure to disinfectants was prospectively associated with a higher risk of developing COPD. Self-reported cleaning/disinfection tasks and exposure to several specific disinfectants evaluated by a JTEM, including glutaraldehyde, bleach, hydrogen peroxide, and alcohol and quaternary ammonium compounds, were associated with a 25% to 38% increased risk of COPD incidence.

To our knowledge, this is the largest prospective study to date to investigate the association of exposure to cleaning products and disinfectants with COPD incidence. Two large cross-sectional studies in Europe (of 13 499 and 502 649 participants) have reported an increased risk of self-reported^17^ or spirometry-defined^16^ COPD, respectively, among professional cleaners. In a cross-sectional study of US working adults, the highest prevalence of self-reported COPD was found among health care support occupations.^28^ In 2018, a prospective analysis of 6235 participants in the European Community Respiratory Health Survey who were followed up for 20 years further showed that women cleaning at home or working as cleaners had accelerated decline in lung function, independently of asthma.^9^ These recent findings confirmed older and smaller analyses of population-based studies reporting increased risk of COPD or chronic bronchitis among cleaners or health care–related professions.^29,30^ Thus, our longitudinal results are consistent with the few data available in the literature and strengthen evidence of a role of exposure to disinfectants in COPD pathogenesis.

In addition, our study extends previous findings by examining disinfection tasks and exposure to specific chemicals among health care workers in relation to COPD. This question is of particular importance to provide guidance for the development of prevention strategies.^20^ We found that use of several specific disinfectants was associated with higher risk of COPD development; these included hypochlorite bleach (chlorine), hydrogen peroxide, alcohol, and quaternary ammonium compounds (commonly used for low-level disinfection of noncritical items, such as environmental surfaces) and glutaraldehyde (used for high-level disinfection).^31^ Several of these exposures often occurred concurrently, and disentangling the role of each product was challenging. When studying combinations of exposure to specific disinfectants, we found the highest risks of COPD incidence among nurses exposed to hypochlorite bleach or hydrogen peroxide and in those combining these exposures with exposure to aldehydes. Both the chemical properties of specific products and the greater number of products used could explain these elevated risks. Moreover, all of the agents that were associated with COPD incidence when evaluated separately have been described as airway irritants.^31,32,33,34^ Inhalation of irritant chemicals may cause injury of the airway epithelium and oxidative stress and may be associated with neutrophilic inflammation.^35,36,37^ Oxidative stress is now recognized to have an important role in COPD pathogenesis and to partly result from environmental exposures. In addition, neutrophilic inflammation is seen in most patients with COPD and correlates with disease severity.^38^ It is notable that exposure to enzymatic cleaners, which have previously been associated with asthma outcomes but are known to contain sensitizers rather than irritants, was not associated with COPD.^14^ Although the associations between exposure to specific disinfectants and COPD incidence must be replicated in independent cohorts and underlying pathophysiological mechanisms must be clarified, there is a biological plausibility that long-term exposure to irritant disinfectants and cleaning agents could contribute to persistent airway damage and COPD pathogenesis.

### Strengths and Limitations

Major strengths of our study include the large population, longitudinal design, and use of a nurse-specific JTEM to evaluate occupational exposure. Previous studies were limited in exposure assessment because they relied only on job titles or self-reported use of general products (sprays or other cleaning products). To evaluate specific exposures in large cohorts, the use of job-exposure matrices is generally favored over self-report.^39^ In this population of nurses, we further showed that using a JTEM, which assigns exposure level based not only on nursing job types but also on disinfection tasks, provides better exposure estimates than a job-exposure matrix by reducing exposure misclassification.^14,24^ Nursing job types and disinfection tasks were self-reported; however because associations were evaluated prospectively (ie, exposure was evaluated before the report of COPD diagnosis), differential recall bias is unlikely. Moreover, we observed dose-response associations for both self-reported cleaning/disinfection tasks and specific exposures evaluated by the JTEM.

Nonetheless, our study had some limitations with regard to exposure assessment. First, the JTEM only evaluated exposure to 7 major products used in US health care settings; exposure to less common products (eg, ortho-phthalaldehyde, peracetic acid, acetic acid, ammonia, phenolics, ethylene oxide, chloramine-T, and “green” products) could not be assessed with this method.^24^ Although a previous study using NHSII data found that fewer than 10% of nurses used these products weekly,^24^ their potential association with COPD development should be examined in future research. Second, we did not collect information regarding the use of protective equipment during cleaning/disinfection tasks in this large population. However, in a substudy on occupational exposures conducted among NHSII nurses with asthma in 2014, 5% of the participants reported using a face mask or other respiratory protection devices when working with disinfectants,^14^ and the use of respiratory protections did not modify the association between disinfectant exposure and poor asthma control. Third, data regarding complete occupational exposure history were not available; indeed, detailed assessment of disinfectant exposure has only been available since 2009. A previous analysis of job changes across 22 years among NHSII participants showed that nurses tend to move to jobs with lower exposure to disinfectants over time.^23^ We therefore expect that women who, after more than 30 years working as a nurse, were still exposed to disinfectants in 2009 had already accumulated a long history of exposure. However, we may have underestimated the association between disinfectant exposures and COPD development, because the reference group (nurses currently nonexposed) likely includes women who had been exposed in the past. Fourth, despite the prospective design and exclusion of all women with COPD before baseline, we cannot exclude the possibility that our results may be subject to a healthy worker effect. Indeed, women with respiratory conditions or symptoms that sometimes precede COPD development, such as asthma, may have left exposed jobs before a COPD diagnosis. A previous study of NHSII participants found that women with a history of asthma were more likely to move to jobs involving a lower level of disinfectant exposure during follow-up.^23^ Thus, the association between disinfectant exposure and COPD incidence may have been underestimated. Despite this potential bias, significant associations between disinfectant exposures and COPD incidence were found among women both with and without a history of asthma.

Another potential limitation was that COPD was defined by questionnaire because lung function measurements were not available in this large, nationwide cohort.^26^ However, a previous validation study showed that self-report of physician-diagnosed COPD was a valid marker of medical record evidence of COPD in the Nurses’ Health Study, a related cohort of health professionals (nurses).^25^ Despite reduced power, sensitivity analyses using a more stringent COPD definition confirmed the main findings. In addition, the most likely source of misclassification of COPD cases is asthma misdiagnosis^25^; however, results were confirmed after excluding all participants with a history of asthma. Finally, although COPD diagnosis in clinical practice relies in large part on spirometry, it has been argued that, in epidemiologic studies, spirometry-based definitions may not capture all COPD phenotypes^40^ because the disease is increasingly recognized as heterogeneous. Studies with different COPD assessment methods may therefore be useful for a more comprehensive understanding of risk factors. It is thus noteworthy that our findings are consistent with a previous report of an association between cleaning work and spirometry-defined COPD.^16^

Reports of an association between cleaning exposures, in particular when evaluated based on job title, and COPD in general population studies may raise concerns regarding potential residual confounding by socioeconomic status.^9^ Investigating this association within a population of registered nurses, who likely have a relatively homogeneous education level and socioeconomic status, provides better control for this type of confounding. Moreover, we have carefully addressed potential confounding by smoking^5^ by adjusting all models for smoking status and pack-years (time-varying variables) and in analyses stratified by smoking status, which confirmed the finding both among smokers and nonsmokers. Nonetheless, our study population of mainly non-Hispanic white female nurses may limit the generalizability of our results. Additional studies in different populations, including other health care–related professions,^41^ among male participants, and among more diverse racial and ethnic groups,^30^ are thus needed. Studies collecting information on both occupational and domestic exposure to cleaning products/disinfectants^9^ would also be helpful to assess the overall association of these exposures with COPD.

## Conclusions

Our findings provide further evidence of an adverse association between disinfectants and cleaning products and respiratory health. A large body of evidence already supports an association between these exposures and asthma^13^; our additional findings of an association with COPD incidence urges the need for the development of exposure-reduction strategies that remain compatible with infection control in health care settings.^19,20^ We estimated that the population-attributable fraction of weekly use of disinfectants on COPD risk among female nurses was 12%; this findings suggests an important contribution of occupational exposure to disinfectants to the burden of COPD among health care workers. Because our study and previous reports^13^ found that a wide range of disinfectants/cleaning agents may be associated with poor respiratory outcomes, the development of new approaches to maintain infection control standards in health care settings may be needed.^20^ Potential safer alternatives include emerging nonchemical technologies for disinfection (eg, steam, UV light) or green cleaning,^20,42,43^ which should be further investigated. Further research is needed to better establish a causal link between exposure to cleaning products and disinfectants and COPD development. Nonetheless, clinicians should be aware of this new risk factor and systematically look for sources of exposure to cleaning products and disinfectants in addition to other occupational exposures in patients with COPD.

## Notes

Supplement. **eAppendix.** Supplemental Methods **References.** **eFigure.** Flow Chart of the Study Population **eTable 1.** Prospective Association Between Self-reported Cleaning/Disinfection Tasks and COPD Incidence in US Female Nurses, Sensitivity Analyses **eTable 2.** Prospective Association Between Self-reported Cleaning/Disinfection Tasks and COPD Incidence in US Female Nurses, According to Smoking Status **eTable 3.** Prospective Association Between Self-reported Cleaning/Disinfection Tasks and COPD Incidence in US Female Nurses, According to Asthma Status **eTable 4.** Prospective Association Between Self-reported Cleaning/Disinfection Tasks and COPD Incidence in US Female Nurses, Using a More Stringent Case Definition **eTable 5.** Prospective Associations Between Combination of Specific Disinfectants/Cleaning Products Evaluated by the JTEM and COPD Incidence in US Female Nurses

## References

[1] GBD 2016 Disease and Injury Incidence and Prevalence Collaborators Global, regional, and national incidence, prevalence, and years lived with disability for 328 diseases and injuries for 195 countries, 1990-2016: a systematic analysis for the Global Burden of Disease Study 2016. Lancet. 2017;390(10100):-. doi:10.1016/S0140-6736(17)32154-2 28919117 PMC5605509

[2] Vogelmeier CF, Criner GJ, Martinez FJ, Global Strategy for the Diagnosis, Management, and Prevention of Chronic Obstructive Lung Disease 2017 report: GOLD executive summary. Am J Respir Crit Care Med. 2017;195(5):557-582. doi:10.1164/rccm.201701-0218PP 28128970

[3] Omland O, Würtz ET, Aasen TB, Occupational chronic obstructive pulmonary disease: a systematic literature review. Scand J Work Environ Health. 2014;40(1):19-35. doi:10.5271/sjweh.3400 24220056

[4] Lytras T, Kogevinas M, Kromhout H, Occupational exposures and 20-year incidence of COPD: the European Community Respiratory Health Survey. Thorax. 2018;73(11):1008-1015. doi:10.1136/thoraxjnl-2017-211158 29574416

[5] Blanc PD, Torén K COPD and occupation: resetting the agenda. Occup Environ Med. 2016;73(6):357-358. doi:10.1136/oemed-2015-103300 27084077

[6] Heederik D, Mannino DM COPD at work: exposures are different than in the past, but still matter. Thorax. 2018;73(11):997-998. doi:10.1136/thoraxjnl-2018-211661 29921700

[7] Schyllert C, Rönmark E, Andersson M, Occupational exposure to chemicals drives the increased risk of asthma and rhinitis observed for exposure to vapours, gas, dust and fumes: a cross-sectional population-based study. Occup Environ Med. 2016;73(10):663-669. doi:10.1136/oemed-2016-103595 27466615

[8] Alif SM, Dharmage SC, Benke G, Occupational exposure to pesticides are associated with fixed airflow obstruction in middle-age. Thorax. 2017;72(11):990-997. doi:10.1136/thoraxjnl-2016-209665 28687678

[9] Svanes Ø, Bertelsen RJ, Lygre SHL, Cleaning at home and at work in relation to lung function decline and airway obstruction. Am J Respir Crit Care Med. 2018;197(9):1157-1163. doi:10.1164/rccm.201706-1311OC 29451393

[10] Marbac M, Sedki M, Boutron-Ruault MC, Dumas O Patterns of cleaning product exposures using a novel clustering approach for data with correlated variables. Ann Epidemiol. 2018;28(8):563-569.e6. doi:10.1016/j.annepidem.2018.05.004 29937403

[11] Dumas O, Wiley AS, Henneberger PK, Determinants of disinfectant use among nurses in U.S. healthcare facilities. Am J Ind Med. 2017;60(1):131-140. doi:10.1002/ajim.22671 27862135 PMC5154899

[12] Wiszniewska M, Walusiak-Skorupa J Occupational allergy: respiratory hazards in healthcare workers. Curr Opin Allergy Clin Immunol. 2014;14(2):113-118. doi:10.1097/ACI.0000000000000039 24451912

[13] Folletti I, Siracusa A, Paolocci G Update on asthma and cleaning agents. Curr Opin Allergy Clin Immunol. 2017;17(2):90-95. doi:10.1097/ACI.0000000000000349 28141626

[14] Dumas O, Wiley AS, Quinot C, Occupational exposure to disinfectants and asthma control in US nurses. Eur Respir J. 2017;50(4):1700237. doi:10.1183/13993003.00237-2017 28982772 PMC5702691

[15] Dumas O, Varraso R, Boggs KM, Association of hand and arm disinfection with asthma control in US nurses. Occup Environ Med. 2018;75(5):378-381. doi:10.1136/oemed-2017-104740 29475850 PMC5899017

[16] De Matteis S, Jarvis D, Hutchings S, Occupations associated with COPD risk in the large population-based UK Biobank cohort study. Occup Environ Med. 2016;73(6):378-384. doi:10.1136/oemed-2015-103406 27001997

[17] Svanes Ø, Skorge TD, Johannessen A, Respiratory health in cleaners in Northern Europe: is susceptibility established in early life? PLoS One. 2015;10(7):e0131959. doi:10.1371/journal.pone.0131959 26168149 PMC4500550

[18] Van den Borre L, Deboosere P Health risks in the cleaning industry: a Belgian census-linked mortality study (1991-2011). Int Arch Occup Environ Health. 2018;91(1):13-21. doi:10.1007/s00420-017-1252-9 28808790

[19] Cummings KJ, Virji MA The long-term effects of cleaning on the lungs. Am J Respir Crit Care Med. 2018;197(9):1099-1101. doi:10.1164/rccm.201801-0138ED 29474796 PMC6726387

[20] Quinn MM, Henneberger PK, Braun B, ; National Institute for Occupational Safety and Health (NIOSH), National Occupational Research Agenda (NORA) Cleaning and Disinfecting in Healthcare Working Group Cleaning and disinfecting environmental surfaces in health care: toward an integrated framework for infection and occupational illness prevention. Am J Infect Control. 2015;43(5):424-434. doi:10.1016/j.ajic.2015.01.029 25792102

[21] Camargo CA Jr, Weiss ST, Zhang S, Willett WC, Speizer FE Prospective study of body mass index, weight change, and risk of adult-onset asthma in women. Arch Intern Med. 1999;159(21):2582-2588. doi:10.1001/archinte.159.21.2582 10573048

[22] Bao Y, Bertoia ML, Lenart EB, Origin, methods, and evolution of the three Nurses’ Health Studies. Am J Public Health. 2016;106(9):1573-1581. doi:10.2105/AJPH.2016.303338 27459450 PMC4981810

[23] Dumas O, Varraso R, Zock JP, Asthma history, job type and job changes among US nurses. Occup Environ Med. 2015;72(7):482-488. doi:10.1136/oemed-2014-102547 25713153 PMC4472505

[24] Quinot C, Dumas O, Henneberger PK, Development of a job-task-exposure matrix to assess occupational exposure to disinfectants among US nurses. Occup Environ Med. 2017;74(2):130-137. doi:10.1136/oemed-2016-103606 27566782 PMC5237395

[25] Barr RG, Herbstman J, Speizer FE, Camargo CA Jr Validation of self-reported chronic obstructive pulmonary disease in a cohort study of nurses. Am J Epidemiol. 2002;155(10):965-971. doi:10.1093/aje/155.10.965 11994237

[26] Varraso R, Chiuve SE, Fung TT, Alternate Healthy Eating Index 2010 and risk of chronic obstructive pulmonary disease among US women and men: prospective study. BMJ. 2015;350:h286. doi:10.1136/bmj.h286 25649042 PMC4707519

[27] Mansournia MA, Altman DG Population attributable fraction. BMJ. 2018;360:k757. doi:10.1136/bmj.k757 29472187

[28] Doney B, Hnizdo E, Syamlal G, Prevalence of chronic obstructive pulmonary disease among US working adults aged 40 to 70 years: National Health Interview Survey data 2004 to 2011. J Occup Environ Med. 2014;56(10):1088-1093. doi:10.1097/JOM.0000000000000232 25285832 PMC4555867

[29] Mirabelli MC, London SJ, Charles LE, Pompeii LA, Wagenknecht LE Occupation and three-year incidence of respiratory symptoms and lung function decline: the ARIC Study. Respir Res. 2012;13(1):24. doi:10.1186/1465-9921-13-24 22433119 PMC3352304

[30] Hnizdo E, Sullivan PA, Bang KM, Wagner G Airflow obstruction attributable to work in industry and occupation among U.S. race/ethnic groups: a study of NHANES III data. Am J Ind Med. 2004;46(2):126-135. doi:10.1002/ajim.20042 15273964

[31] Hawley B, Casey M, Virji MA, Cummings KJ, Johnson A, Cox-Ganser J Respiratory symptoms in hospital cleaning staff exposed to a product containing hydrogen peroxide, peracetic acid, and acetic acid. Ann Work Expo Health. 2017;62(1):28-40. doi:10.1093/annweh/wxx087 29077798 PMC5757516

[32] Quirce S, Barranco P Cleaning agents and asthma. J Investig Allergol Clin Immunol. 2010;20(7):542-550. 21313993

[33] Ernstgård L, Sjögren B, Johanson G Acute effects of exposure to vapors of hydrogen peroxide in humans. Toxicol Lett. 2012;212(2):222-227. doi:10.1016/j.toxlet.2012.05.025 22677343

[34] Gonzalez M, Jégu J, Kopferschmitt M-C, Asthma among workers in healthcare settings: role of disinfection with quaternary ammonium compounds. Clin Exp Allergy. 2014;44(3):393-406. doi:10.1111/cea.12215 24128009

[35] Dumas O, Matran R, Zerimech F, Occupational exposures and fluorescent oxidation products in 723 adults of the EGEA study. Eur Respir J. 2015;46(1):258-261. doi:10.1183/09031936.00177614 25837036

[36] Tarlo SM, Lemiere C Occupational asthma. N Engl J Med. 2014;370(7):640-649. doi:10.1056/NEJMra1301758 24521110

[37] McGovern TK, Goldberger M, Allard B, Neutrophils mediate airway hyperresponsiveness after chlorine-induced airway injury in the mouse. Am J Respir Cell Mol Biol. 2015;52(4):513-522. doi:10.1165/rcmb.2013-0430OC 25192041

[38] Barnes PJ Inflammatory mechanisms in patients with chronic obstructive pulmonary disease. J Allergy Clin Immunol. 2016;138(1):16-27. doi:10.1016/j.jaci.2016.05.011 27373322

[39] Loomis D Towards population-wide exposure assessment. Occup Environ Med. 2012;69(7):455-456. doi:10.1136/oemed-2012-100928 22685042

[40] Bakke PS, Rönmark E, Eagan T, ; European Respiratory Society Task Force Recommendations for epidemiological studies on COPD. Eur Respir J. 2011;38(6):1261-1277. doi:10.1183/09031936.00193809 22130763

[41] Su FC, Friesen MC, Humann M, Clustering asthma symptoms and cleaning and disinfecting activities and evaluating their associations among healthcare workers. Int J Hyg Environ Health. 2019;222(5):873-883. doi:10.1016/j.ijheh.2019.04.001 31010790 PMC6883647

[42] Garza JL, Cavallari JM, Wakai S, Traditional and environmentally preferable cleaning product exposure and health symptoms in custodians. Am J Ind Med. 2015;58(9):988-995. doi:10.1002/ajim.22484 26040239 PMC4976595

[43] Goodyear N, Markkanen P, Beato-Melendez C, Cleaning and disinfection in home care: a comparison of 2 commercial products with potentially different consequences for respiratory health. Am J Infect Control. 2018;46(4):410-416. doi:10.1016/j.ajic.2017.09.033 29169933

