# Supplementary material for: Association of Occupational Exposure to Disinfectants With Incidence of Chronic Obstructive Pulmonary Disease Among US Female Nurses
Source: JAMA Netw Open. 2019 Oct 18;2(10):e1913563. doi: 10.1001/jamanetworkopen.2019.13563 (PMC6813668; doi:10.1001/jamanetworkopen.2019.13563)
Supplement: Supplement. — eAppendix. Supplemental Methods References. eFigure. Flow Chart of the Study Population eTable 1. Prospective Association Between Self-reported Cleaning/Disinfection Tasks and COPD Incidence in US Female Nurses, Sensitivity Analyses eTable 2. Prospective Association Between Self-reported Cleaning/Disinfection Tasks and COPD Incidence in US Female Nurses, According to Smoking Status eTable 3. Prospective Association Between Self-reported Cleaning/Disinfection Tasks and COPD Incidence in US Female Nurses, According to Asthma Status eTable 4. Prospective Association Between Self-reported Cleaning/Disinfection Tasks and COPD Incidence in US Female Nurses, Using a More Stringent Case Definition eTable 5. Prospective Associations Between Combination of Specific Disinfectants/Cleaning Products Evaluated by the JTEM and COPD Incidence in US Female Nurses [file jamanetwopen-2-e1913563-s001.pdf]

Supplementary Online Content

Dumas O, Varraso R, Boggs KM, et al. Association of occupational exposure to disinfectants with incidence of chronic obstructive pulmonary disease among US female nurses. *JAMA Netw Open*. 2019;2(10):e1913563. doi:10.1001/jamanetworkopen.2019.13563

eAppendix. Supplemental Methods

eReferences.

eFigure. Flow Chart of the Study Population

eTable 1. Prospective Association Between Self-reported Cleaning/Disinfection Tasks and COPD Incidence in US Female Nurses, Sensitivity Analyses

eTable 2. Prospective Association Between Self-reported Cleaning/Disinfection Tasks and COPD Incidence in US Female Nurses, According to Smoking Status

eTable 3. Prospective Association Between Self-reported Cleaning/Disinfection Tasks and COPD Icidence in US Female Nurses, According to Asthma Status

eTable 4. Prospective Association Between Self-reported Cleaning/Disinfection Tasks and COPD Incidence in US Female Nurses, Using a More Stringent Case Definition

eTable 5. Prospective Associations Between Combination of Specific Disinfectants/Cleaning Products Evaluated by the JTEM and COPD Incidence in US Female Nurses

This supplementary material has been provided by the authors to give readers additional information about their work.

eAppendix. Supplemental Methods

Occupational exposure to disinfectants

The occupational questionnaire used in our study (2009, 2011, 2013) was adapted to U.S. context from questionnaires used in European studies (European Community Respiratory Health Survey[1] and Epidemiological study on the Genetics and Environment of Asthma),[2] with additions of relevant tasks based on results from a study of asthma among healthcare workers in Texas.[3]

Job type and tasks

Current nursing job type was evaluated using the question "Which best describes your current employment status?", in 8 categories: nursing in the emergency room, operating room, intensive care unit, other inpatient nurse, outpatient or community, other hospital nursing, nursing outside hospital and nursing education or administration. The two questions regarding the frequency (days/week) of disinfection tasks were: 'Thinking about your current job and the use of disinfectants (such as ethylene oxide, hydrogen peroxide, orthophthalaldehyde, formaldehyde, glutaraldehyde and bleach): (a) On how many days per week, on average, do you clean medical instruments with disinfectants? (b) On how many days per week, on average, do you clean surfaces (like floors, tables) at work with disinfectants? (never, <1 day/week, 1-3 days/week, 4-7 days/week)". A specific question was asked about the use of sprays: "In your current job, on how many days per week, on average, do you use spray or aerosol products? (never, <1 day/week, 1-3 days/week, 4-7 days/week)".

For each question, participants responding "1-3 days/week" or "4-7 days / week" were classified as "weekly use" and those responding "never" or "<1 day/week" as "less than weekly". The two questions on disinfecting tasks (to clean medical instruments/to clean surfaces) were combined to create a 3-level variable as previously,[4,5] to define tasks performed weekly: no weekly disinfection tasks; weekly use of disinfectants to clean surfaces only; and weekly use of disinfectants to clean at least medical instruments (regardless of the use of disinfectants to clean surfaces).The category 'clean instruments only' was not studied separately because of the low number of NHSII nurses in this category (2.9%).[5]

Job-task exposure matrix (JTEM) to evaluate exposure to specific disinfectants

Exposure to seven major disinfectants/cleaning products was evaluated by the JTEM (formaldehyde, glutaraldehyde, hypochlorite bleach, hydrogen peroxide, alcohol [ethanol, methanol, isopropanol], quats, and enzymatic cleaners). The development of the JTEM has been described in detail elsewhere.[5] Briefly, it used the responses to the questions described above in a population of 9,073 nurses without asthma (2014-2015), drawn from the Nurses' Health Study II. We generated the JTEM based on the percentage of participants reporting exposure to a given disinfectant for a given nursing job and task category. The "job-task" axis of the JTEM included the 24 possible combinations of 8 types of nursing jobs by 3 categories of cleaning tasks (surfaces only, at least instruments, none). Specific cut-offs were defined to classify exposure in "low", "medium" and "high" levels for each disinfectant, in a given job or job-task combination.

Given the way the JTEM was designed, "high level" of exposure should be understood as higher probability of frequent (weekly) exposure. As the cut-offs chosen were disinfectant-specific, and were relative to the average level of use within each job/task, "high level" is disinfectant specific (e.g., "high" level of glutaraldehyde does not compare to "high" level of exposure to alcohol in terms of intensity, frequency or concentration of exposure).

To study combinations of specific products evaluated by the JTEM, we classified exposure to products found associated with COPD when studied separately into 5 mutually exclusive categories: low exposure level for all 7 products evaluated by the JTEM (reference); high exposure level to alcohol or quats only; high exposure level to aldehydes (formaldehyde or glutaraldehyde); high exposure level to hypochlorite bleach or hydrogen peroxide; and high exposure level to both aldehydes and hypochlorite bleach/hydrogen peroxide (eTable 5).

COPD

In all biennial questionnaires since 1989, information on physician-diagnosed COPD was collected, first through report of "major illnesses" in a free-text field, and since 1999 by a specific question on physician-diagnosed chronic bronchitis or emphysema (eFigure 1). In our analyses, incident cases of clinician-diagnosed COPD were identified from 2009 to 2015. All women with any history of COPD at baseline (2009) were excluded (eFigure1).

The COPD stringent case definition, used in sensitivity analyses, was based on responses to a supplemental questionnaire on COPD, sent between 2015 and 2017 to all participants who ever reported clinician-diagnosed emphysema or chronic bronchitis in biennial questionnaires (responses collected until July 2018, response rate=76%). The specific questionnaire gathered, among other data, information confirming a physician's diagnosis of emphysema, chronic bronchitis, or COPD, as well as the dates of symptom onset and

diagnosis. In the current study, the stringent case definition included participants who reiterated on the supplemental questionnaire that a physician had diagnosed her as having chronic bronchitis, emphysema, or COPD. A validation study was previously conducted in the Nurses' Health Study, a related cohort of US nurses, to evaluate this epidemiologic definition of COPD.[6] Briefly, in a random sample of participants who reported COPD, we obtained medical records (two pulmonary function tests, two chest films, one chest computed tomography scan, two office or emergency department visits for COPD or asthma, and one hospital discharge summary regarding COPD or asthma), and a physician reviewed them in a blinded fashion. The validation study confirmed 83% of the nurses' reports of newly diagnosed COPD based on this definition.[6]

Asthma

In all biennial questionnaires since 1991, the participants were asked if they had physician-diagnosed asthma. Supplemental questionnaires on asthma were sent in 1998, 2003 and 2014, to all living women who had reported a physician diagnosis of asthma in earlier biennial questionnaire(s). More detailed information on dates of symptom onset and diagnosis, asthma symptoms, medications and hospitalizations for asthma were collected. Asthma cases (definition 1) were defined as participants who reiterated on at least one of the supplemental questionnaires that they had physician-diagnosed asthma, and reported using an asthma medication since diagnosis.[7]

Race/ethnicity

Race and ethnicity were considered as potential confounders in our analyses.[3] Race and ethnicity were evaluated by questionnaire (self-report). In the NHSII 1989 questionnaire, participants were asked to report their major ancestry ("Southern European / Meditarranean", "Scandinavian", "Other Caucasian", "African-American", "Hispanic", "Asian" or "Other"). Race/ethnicity was further evaluated in the 2005 questionnaire, asking participants "Do you consider yourself to be Spanish/Hispanic/Latina?" ("No"/"Yes") and "Which categories best describe your race?" ("White", "Asian", "Black or African American", "American Indian/Alaska Native", "Native Hawaiian or Pacific Islander", or "Other"). Because the NHSII population is in large majority white and non-Hispanic, race was studied in three categories ("White", "Black", "Other") and ethnicity in two categories ("Hispanic", "non-Hispanic").

eReferences

1. Mirabelli MC, Zock JP, Plana E, et al. Occupational risk factors for asthma among nurses and related healthcare professionals in an international study. *Occup Env Med*. 2007;64(7):474-479.
2. Donnay C, Denis M-A, Magis R, et al. Under-estimation of self-reported occupational exposure by questionnaire in hospital workers. *Occup Env Med*. 2011;68(8):611-617.
3. Delclos GL, Gimeno D, Arif AA, et al. Occupational risk factors and asthma among health care professionals. *Am J Respir Crit Care Med*. 2007;175(7):667-675.
4. Dumas O, Wiley AS, Quinot C, et al. Occupational exposure to disinfectants and asthma control in US nurses. *Eur Respir J*. 2017;50(4):pii: 1700237.
5. Quinot C, Dumas O, Henneberger PK, et al. Development of a job-task-exposure matrix to assess occupational exposure to disinfectants among US nurses. *Occup Env Med*. 2017;74(2):130-137.
6. Barr RG, Herbstman J, Speizer FE, Camargo CA Jr. Validation of self-reported chronic obstructive pulmonary disease in a cohort study of nurses. *Am J Epidemiol*. 2002;155(10):965-971.
7. Camargo CA Jr, Weiss ST, Zhang S, Willett WC, Speizer FE. Prospective study of body mass index, weight change, and risk of adult-onset asthma in women. *Arch Intern Med*. 1999;159(21):2582-2588.

eFigure. Flow chart of the study population. NHSII: Nurses' Health Study II.

[a] Participants with missing data for occupational exposure were older (mean age: 61 years) than included participants (mean age: 55 years p<0.001), but did not differ from included participants for smoking status, body mass index, race, ethnicity or COPD diagnosis.

[b] Participants with missing data for pack-years of smoking were less often white (93%) than included participants (96%, p=0.03), but did not differ from included participants for age, body mass index, ethnicity, self-reported use of disinfectants or COPD diagnosis.

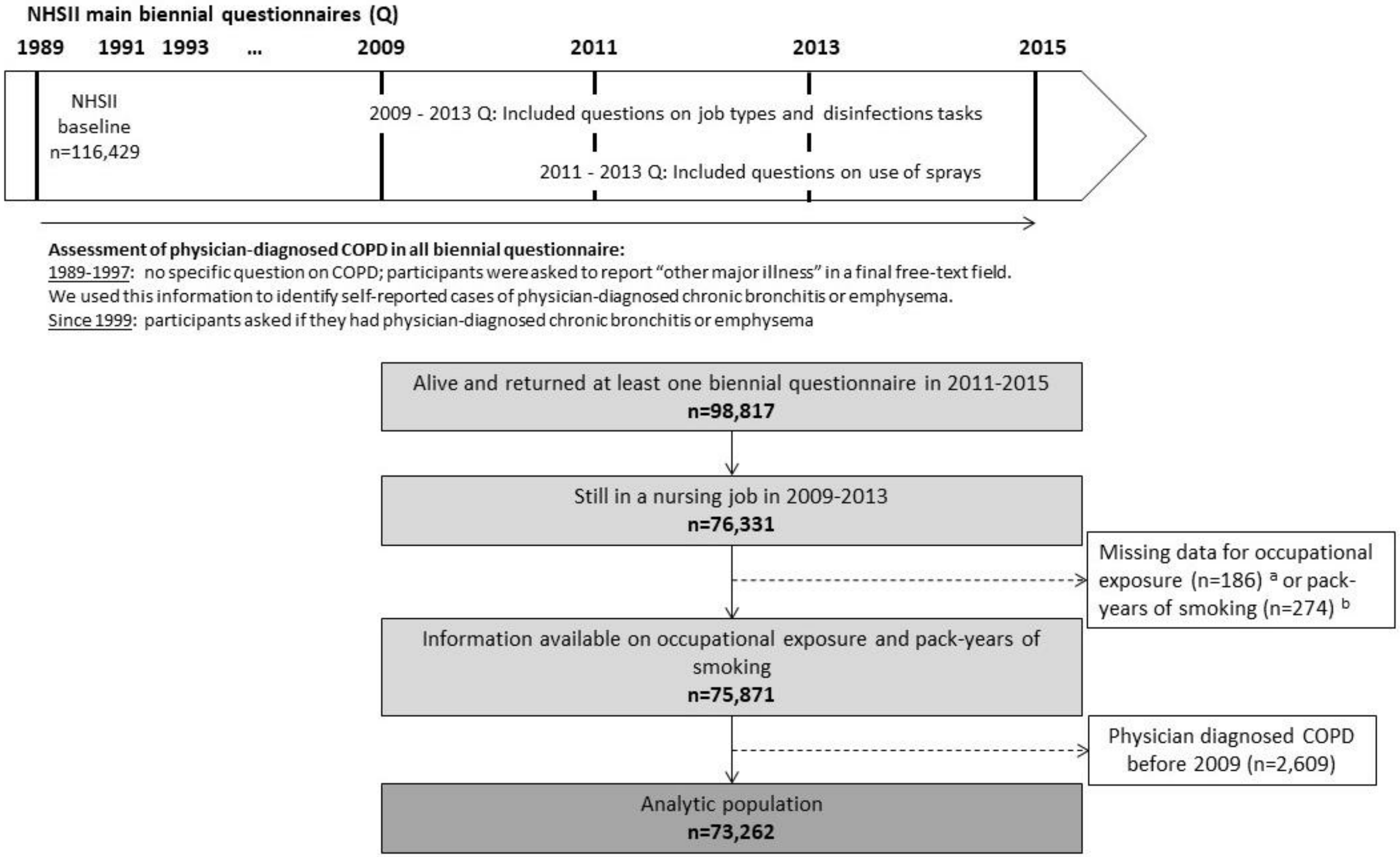

**eTable 1. Prospective association between self-reported cleaning/disinfection tasks and COPD incidence in US female nurses, sensitivity analyses**						
Occupational exposure [a]	Model further adjusted for pack-years^2 of smoking [b]		Model further adjusted for diet quality [b, c]		Model excluding participants with previous comorbidities[b,d]	
	HR	95% CI	HR	95% CI	HR	95% CI
Weekly use of disinfectant						
None (ref.)	1	-	1	-		
Any disinfectant	1.34	1.14-1.59	1.33	1.13-1.57	1.27	1.07-1.50
Surface only	1.38	1.13-1.68	1.37	1.13-1.67	1.30	1.06-1.60
Instruments	1.31	1.06-1.60	1.29	1.05-1.58	1.23	0.99-1.52

Abbreviations: HR, hazard ratio; CI, confidence interval.

[a] Exposure evaluated as the highest exposure level at any of the questionnaire cycles before time of diagnosis.

[b] All models were adjusted for age, smoking status and pack-years (continuous), race, ethnicity and body mass index.

[c] Diet quality measured by the Alternate Healthy Eating Index 2010 (AHEI-2010), divided into quintiles.

[d] Analyses excluding cases of cardiovascular diseases and cancer. Analyses conducted in 346,529 person-years (547 incident COPD cases).

eTable 2. Prospective association between self-reported cleaning/disinfection tasks and COPD incidence in US female nurses, according to smoking status

Occupational exposure [a]	Never smoker (245,005 person-years, 238 COPD cases)		Ever smoker (123,124 person-years, 344 COPD cases)		P interaction
	HR	95% CI	HR	95% CI	
Weekly use of disinfectant					
None (ref.)	1	-	1	-	
Any disinfectant	1.34	1.03-1.73	1.38	1.11-1.71	0.91
Surface only	1.28	0.94-1.74	1.47	1.14-1.91	0.48
Instruments	1.40	1.02-1.92	1.28	0.97-1.68	0.58

Abbreviations: HR, hazard ratio; CI, confidence interval.
[a] Models were adjusted for age, race and body mass index (models were not adjusted for ethnicity because of low numbers in stratified analysis); and for pack-years (continuous) of smoking among ever smokers. Exposure evaluated as the highest exposure level at any of the questionnaire cycles before time of diagnosis.

eTable 3. Prospective association between self-reported cleaning/disinfection tasks and COPD incidence in US female nurses, according to asthma status

Occupational exposure [a]	Never asthma (301,313 person-years, 283 COPD cases)		Ever asthma[a] (50,299 person-years, 190 COPD cases)		P interaction
	HR	95% CI	HR	95% CI	
Weekly use of disinfectant					
None (ref.)	1	-	1	-	
Any disinfectant	1.31	1.03-1.66	1.37	1.02-1.84	0.78
Surface only	1.49	1.13-1.97	1.20	0.83-1.73	0.38
Instruments	1.12	0.82-1.52	1.56	1.10-2.23	0.16

Abbreviations: HR, hazard ratio; CI, confidence interval.

[a] Models were adjusted for age, smoking status and pack-years (continuous), race, ethnicity and body mass index. Exposure evaluated as the highest exposure level at any of the questionnaire cycles before time of diagnosis.

[b] Ever asthma before baseline (2009), using case definition 1. Participants who reported asthma in main questionnaire but did not meet asthma case definition 1, or who were diagnosed with asthma after 2009, were excluded from the analyses.

eTable 4. Prospective association between self-reported cleaning/disinfection tasks and COPD incidence in US female nurses, using a more stringent case definition[a]

	Person-years	No. of cases	Age-adjusted HR		Multivariable-adjusted HR	
			HR	95% CI	HR	95% CI
Job type						
Education or administration	52,827	32	1	-	1	-
Outpatient, other nurses	195,946	136	1.13	0.77-1.67	1.17	0.79-1.72
ER or inpatient unit	94,837	69	1.31	0.86-2.00	1.21	0.79-1.85
Operating room	23,928	21	1.54	0.89-2.68	1.47	0.84-2.56
Weekly use of disinfectant						
None (ref.)	183,292	120	1	-	1	-
Any disinfectant	184,245	138	1.25	0.97-1.59	1.24	0.96-1.59
Surface only	93,225	75	1.30	0.97-1.74	1.32	0.99-1.78
Instruments	91,020	63	1.19	0.87-1.61	1.14	0.84-1.56

Abbreviations: HR, hazard ratio; CI, confidence interval; ER, emergency room.

[a] Participants who reiterated that a physician had diagnosed her as having emphysema, chronic bronchitis and/or COPD in the supplemental questionnaire (stringent case definition, n=258).

Multivariable models were adjusted for age, smoking status and pack-years (continuous), race, ethnicity and body mass index.

Exposure evaluated as the highest exposure level at any of the questionnaire cycles before time of diagnosis.

Observations with missing values for pack-years of smoking (<0.5%) were excluded from analyses. Observations with missing value for body mass index (3.7%) were included in the model as a “missing” category.

**eTable 5. Prospective associations between combination of specific disinfectants/cleaning products evaluated by the JTEM and COPD incidence in US female nurses**				
Exposure combinations (JTEM estimates) [a]	Person-years	No. of cases	Multivariable-adjusted HR[c]	
			HR	95% CI
Low exposure level for all products (reference) [b]	111,579	174	1	-
1): High exposure level to alcohol or quats only	11,545	22	1.32	0.84-2.06
2): High exposure level to formaldehyde or glutaraldehydew	25,472	35	1.21	0.84-1.74
3): High exposure level to hypochlorite bleach or hydrogen peroxide	44,466	84	1.58	1.21-2.06
4): 2)+3)	105,099	164	1.36	1.08-1.70

Abbreviations: HR, hazard ratio; CI, confidence interval; JTEM – Job-Task-Exposure Matrix.

[a] Mutually exclusive categories; Exposure evaluated based on the highest exposure level at any of the questionnaire cycles before time of diagnosis.

[b] classified as low exposure level for all seven products evaluated by the JTEM. Observations with missing values for pack-years of smoking (<0.5%) were excluded from analyses.

[c] Multivariable models were adjusted for age, smoking status and pack-years (continuous), race, ethnicity and body mass index. Observations with missing value for body mass index (3.8%) were included in the model as a “missing” category.
